# Magnetic Resonance Microscopy for Assessment of Morphological Changes in Hydrating Hydroxypropylmethylcellulose Matrix Tablets *In Situ*–Is it Possible to Detect Phenomena Related to Drug Dissolution Within the Hydrated Matrices?

**DOI:** 10.1007/s11095-014-1334-2

**Published:** 2014-03-15

**Authors:** Piotr Kulinowski, Anna Młynarczyk, Krzysztof Jasiński, Przemysław Talik, Marco L. H. Gruwel, Bogusław Tomanek, Władysław P. Węglarz, Przemysław Dorożyński

**Affiliations:** 1 Department of Magnetic Resonance Imaging, Institute of Nuclear Physics PAN, ul. Radzikowskiego 152, 31-342 Kraków, Poland; 2 Department of Inorganic and Analytical Chemistry Pharmaceutical Faculty, Medical College, Jagiellonian University, ul. Medyczna 9, 30-688 Kraków, Poland; 3 National Research Council Canada, Institute for Biodiagnostics, 435 Ellice Avenue, Winnipeg, Manitoba Canada R3B 1Y6; 4 Thunder Bay Regional Research Institute, 980 Oliver Road, Thunder Bay, Ontario P7B 6V4 Canada; 5 Department of Pharmaceutical Technology and Biopharmaceutics Pharmaceutical Faculty, Jagiellonian University, ul. Medyczna 9, 30-688 Kraków, Poland

**Keywords:** controlled release (CR), HPMC, hydrophilic matrices, magnetic resonance imaging (MRI), MR T_2_ relaxometry

## Abstract

**Purpose:**

So far, the hydrated part of the HPMC matrix has commonly been denoted as a “gel” or “pseudogel” layer. No MRI-based results have been published regarding observation of internal phenomena related to drug dissolution inside swelling polymeric matrices during hydration. The purpose of the study was to detect such phenomena.

**Methods:**

Multiparametric, spatially and temporally resolved T_2_ MR relaxometry, *in situ*, was applied to study formation of the hydration progress in HPMC matrix tablets loaded with L-dopa and ketoprofen using a 11.7 T MRI system. Two spin-echo based pulse sequences were used, one of them specifically designed to study short T_2_ signals.

**Results:**

Two components in the T_2_ decay envelope were estimated and spatial distributions of their parameters, i.e. amplitudes and T_2_ values, were obtained. Based on the data, different region formation patterns (i.e. multilayer structure) were registered depending on drug presence and solubility. Inside the matrix with incorporated sparingly soluble drug a specific layer formation due to drug dissolution was detected, whereas a matrix with very slightly soluble drug does not form distinct external “gel-like” layer.

**Conclusions:**

We have introduced a new paradigm in the characterization of hydrating matrices using ^1^H MRI methods. It reflects molecular mobility and concentration of water inside the hydrated matrix. For the first time, drug dissolution related phenomena, i.e. particular front and region formation, were observed by MRI methods.

## INTRODUCTION

Studies on the behavior of controlled release (CR) hydroxypropylmethylcellulose (HPMC) matrix systems emerged in the late 1960’s (1). Then, in the mid 90’s the spatial characteristics of the drug loaded matrix during hydration was proposed (2). In this concept, the hydrated polymeric matrix is divided into three zones characterized by different properties that allow their visual identification. The borders between the particular zones were defined as fronts–an erosion front separating the hydrogel from the surrounding solution, a swelling front identified between the hydrated glassy polymer and dry glassy polymer and diffusion front between solid and dissolved drug in the matrix. This early approach to understanding the structure of swelling matrices was based on simple photo imaging of hydrated tablets, where water penetration was restricted by placing the tablet between two glass plates.

The development of noninvasive imaging techniques e.g. magnetic resonance imaging (MRI), attenuated total reflection Fourier transformed infrared microscopy (ATR-FTIR) and others, led to a deeper insight into the structure of swelling HPMC matrix systems (3–5). However, the basic concept of a three front model for the swollen tablet is recognized as a gold standard to describe the structure of hydrated CR matrix systems.

It must be pointed out that in the structural analyses of swelling matrices, the presence of an active substance was not discussed systematically. This is connected to fact that photo imaging techniques are not able to distinguish the influence of colorless substances on the structure of the matrix. Other highly sensitive chemical imaging techniques, e.g. FTIR microscopy, were able to detect changes in the concentration of particular components of the matrix (i.e. polymer, water, drug) rather than its structure. MRI techniques are noninvasive and allow to study model or commercial dosage forms of various shape and size. For this reason most of the studies used MRI as a method to evaluate matrix tablet hydration (6–8). But these techniques are mainly focused on proton imaging, which is not appropriate for the detection of drug molecules within the matrix. In the literature concerning pharmaceutical applications of MRI there are only two examples of MR imaging using fluorine nuclei (9,10), directly imaging the active substance. Due to limitation to fluorine containing drugs, this approach cannot be applied universally.

On the other hand it is without doubt that properties of the active substance present within the CR formulation will modify the behavior of matrix system, which should, in principle, be possible to detect with proton MRI. The MRI studies concerning HPMC matrices loaded with active substances of different solubility are few in number. Kowalczuk *et al.* 2004 studied HPMC tablets with various polymer content (100, 75 and 67%) loaded with tetracycline hydrochloride, a drug highly soluble in water. It was stated that drug loading reduced the integrity and the resistance of network structure against the penetration of water molecules (11). Simultaneously, swelling properties of the polymeric matrix increased. In the work of Tajarobi *et al.* it was shown that soluble substances, e.g. lactose, mannitol etc., increased the hydration rate of HPMC matrices whilst insoluble excipients such as dicalcium phosphate had an inhibiting influence on the water penetration into the matrix (6). Kulinowski *et al.* 2011 proposed an MR image intensity histogram based segmentation analysis of MR spin-echo images of an HPMC matrix with quetiapine fumarate as soluble drug and excipients (12). According to the trimodal image intensity histogram a more composite structure/morphology of the hydrating matrix was revealed. Despite two decades of extensive exploration of this problem, using various imaging methods including MRI, a consistent picture of a hydrating HPMC matrix containing drugs of different solubility has not been presented yet.

In our previous paper we have discussed a novel multiparametric approach to the investigation of hydrated HPMC matrices. It was based on relaxometric data obtained with magnetic resonance microscopy (13). In this work the coexistence of two components in the T_2_ envelope across the fully hydrated polymeric region as well as a multilayer matrix composition was demonstrated. The analysis was carried out on the simplest example of pure HPMC matrix tablets. This paper extends the application of previously developed methodology based on spatially resolved, magnetic resonance microscopy (T_2_ relaxometry) to HPMC matrix loaded with drugs of different solubility.

The goal of the study was to apply the previously developed methodology to detect phenomena related to drug presence in the hydrating HPMC matrices, and in consequence, to compare the multilayer structure of HPMC based systems with drug substances of different solubility.

## MATERIALS AND METHODS

Two drug substances: L-dopa (LD) (Sigma-Aldrich) and ketoprofen (KT) (Sigma-Aldrich) were used as model drugs of different solubility. Solubility difference between drugs was chosen to be within two orders of magnitude in 0.1 M hydrochloric acid solutions. According to the European Pharmacopoeia 6.0, ketoprofen is very slightly soluble in water whilst L-Dopa is slightly soluble in water. In 0.1 M hydrochloric acid solutions L-dopa is sparingly soluble (1 g in 30–100 mL) (14), ketoprofen is very slightly soluble (1 g in 1,000–10,000 mL) (14), its solubility is 0.13 mg/mL (15). HPMC–Metolose 65 SH 400 and 10,000 cP (Shin-Etsu Chemical Co., Ltd. Tokyo, Japan) were used for the preparation of the matrix tablets. The 1:1 (by weight) mixtures of drugs and polymer were prepared by mixing substances in polyethylene bag for 5 min. Tablets, 9 mm in diameter and a height of 3 mm (200 mg) were prepared from mixtures by direct compression using a 10-station ERWEKA TRB 10 press. The hardness of the tablets was in the range 5.4–6.2 kG, the friability was 0.3–0.5%.

### Experimental Protocol

The tablets were kept in 50 mL of hydrochloric acid (0.1 M) at room temperature (~22°C). The tablets were taken for measurements after 30, 60, 120 and 180 min of immersion in hydrochloric acid solution. At these time points the solution was removed and the tablets were protected to minimize the evaporation. The MR measurements were carried out using a home-built dedicated MR probe (*vide infra*). After measurements the samples were disposed. The procedure was repeated for each of the time points. One sample was measured for one hydration time. A total number of 24 independent samples was measured using the presented method–samples at four different hydration times, of two different polymer viscosities and drug loading were measured.

### MRI Data Acquisition

Spatially resolved T_2_ MR relaxometry as described in the preceding paper (13) was applied. The measurements were carried out with a 11.7 T (500 MHz) vertical bore magnet (Oxford Instruments U.K.) equipped with a water-cooled, self-shielded gradient system SGRAD 123/72/S with an inner diameter of 72.5 mm and Avance console (Bruker, Germany) operating under ParaVision 3.0.2 system. A radio-frequency saddle coil (10 mm ID, 10 mm length) was used. For multiparametric T_2_ imaging, two separate sequences were applied. The long time scale of the T_2_ decay envelope (up to 208 ms with resolution of 6.5 ms) was probed using the Multi Slice Multi Echo (MSME) sequence. The short time scale of the T_2_ decay envelope (up to 12 ms with a resolution of 1 ms) was probed using the Broad Line Imaging Package Modes (BLIP_MODES) MRI pulse sequence (ParaVision 3.0.2, Bruker, Germany). The following MSME sequence parameters were applied: TE/TR = 6.5/4,000 ms, number of accumulations = 2, number of images = 32, matrix size = 256 × 256 × 1, slice thickness = 1 mm, field of view = 15 × 15 mm. A BLIP pulse sequence was used with following parameters: TE/TR = 3/500 ms. Number of accumulations = 2, Number of echoes = 10, Matrix size =256 × 256 × 1, Slice thickness = 1 mm, field of view = 15 × 15 mm. The MSME and BLIP data sets were analyzed by custom developed scripts in MATLAB (The MathWorks, Inc.).

As a result, a four-parameter description of each spatial position (pixel) was developed. Details can be found in the article by Kulinowski *et al.* (13).

### Differential Scanning Calorimetry (DSC)

The tablets were kept in similar conditions as used for the MRI experiments and removed from the solution after 2 h of soaking. The samples of the external layer were carefully peeled off and precisely weighted in hermetic aluminum pans (15 μL, up to 3 MPa). Differential scanning calorimetry (DSC) was carried out using the EXSTAR DSC 7020 apparatus (SII NanoTechnology Inc.) equipped with a DSC7020 electric cooling unit and assessed by the Muse Standard Analysis (SII NanoTechnology Inc.) software. The temperature calibration was done with indium and tin (melting temperature Tm of In is 156.6°C, Tm of Tin is 231.88°C). The calibration of enthalpy change was done with Tin (melting enthalpy ΔHm of Tin is 60.46 J/g). The procedure was carried out at 5.0°C/min.

The following thermal protocol was used for cooling/heating experiments: start at 30.0°C; cooling from 30.0 to −50.0°C at 5.0°C/min; equilibrating at −50.0°C for 10 min; heating from −50.0 to 30°C at 5.0°C/min. Flow rate of dynamic nitrogen atmosphere was 50 mL/min.

## RESULTS AND DISCUSSION

Only results for HPMC 10,000 cP based matrices are presented and referred to as HPMC. However, for the HPMC 400 cP based matrices, similar results were obtained. For pure HPMC 400 cP they were shown in the previous article (see Kulinowski *et al.* 2012 (13)). Due to the fact that tendencies in behavior of matrices based on both HPMC types were similar, in this article, these results can be referred to confirm the representativeness of presented findings.

**Fig. 1:**
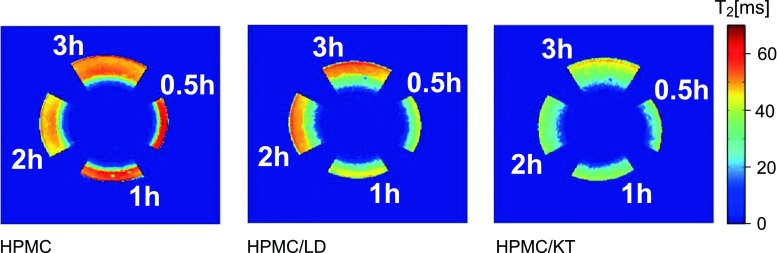
T_2_ maps of the longer T_2_ component of hydrated matrix pie pieces. Each matrix is shown at four hydration times, i.e. 0.5, 1, 2 and 3 h.

Figure 1 presents T_2_ maps of hydrated matrix fragments. Each matrix is shown at four hydration times, i.e. 0.5, 1, 2 and 3 h. The initial, qualitative assessment shows clearly that all three formulations differ at particular time points despite the similar size of the hydrated area of the matrix. At 3 h, a layer containing water of high mobility (T_2_~60 ms) is present in pure HPMC matrices and HPMC with L-Dopa but it is absent in the HPMC matrix loaded with ketoprofen.

**Fig. 2:**
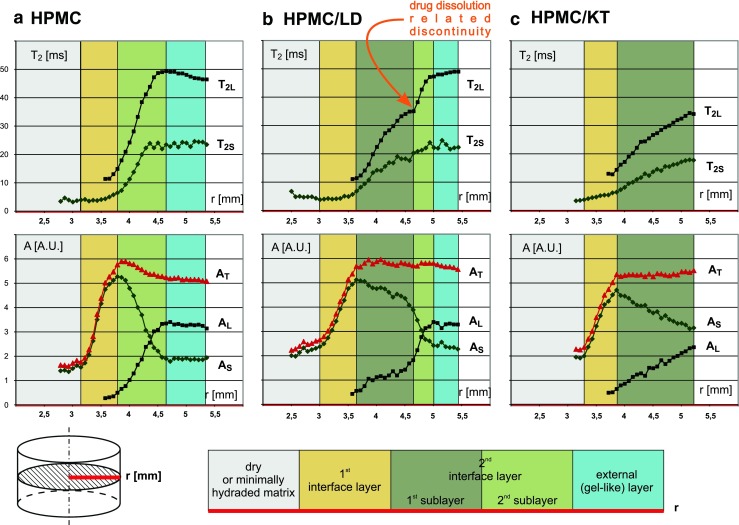
T_2_ values (*top row*) and signal amplitudes (*lower row*) of two components in the T_2_ decay envelope *vs*. tablet radius at 2 h of hydration for HPMC (*left*) HPMC/LD (*centre*) HPMC/KT (*right*) tablets. T_2L_–T_2_ relaxation time of longer component, T_2S_–T_2_ relaxation time of shorter component, A_S_–amplitude of shorter component, A_L_–amplitude of longer component. And A_T_–total amplitude (A_S_ + A_L_), indicated in *red*. Color scheme used: *gray*–minimally hydrated matrix (*solid like*), *yellow*–the first interface layer, *deep/light green*–the second interface layer, *blue*–firm “gel-like” layer.

A more comprehensive description of the systems can be given using radial parameter profiles according to the methodology described in our previous paper (13). It was shown that two types of water can be identified in the hydrated pure HPMC matrix. These proton pools are represented by separate T_2_ components. Each component is characterized by two parameters: a T_2_ time constant and an amplitude referred to as proton density (PD). The approach yields the following four estimated parameters: T_2_ of the shorter component (T_2S_), amplitude of the shorter component (A_S_), T_2_ of the longer component (T_2L_) and amplitude of the longer component (A_L_). This approach also appeared to be valid for matrices loaded with drug substances of different solubility. The T_2_/PD patterns for the investigated matrices differ as shown in Fig. 2.

To identify the characteristics of water occurring within the hydrated matrices, DSC studies were carried out. The results of the DSC measurements will be described in details in the subsequent sections of the paper. However, for clarity it is important to note at this point, that thermal analysis confirmed the absence of free (bulk) water in the samples taken from the external part of the matrices. Consequently, the two components in T_2_/PD profiles can be assigned to two types of freezable bound water, namely: bound water of higher mobility (T_2L_/A_L_) and bound water of lower mobility (T_2S_/A_S_).

One of the most important results obtained from the presented study is the detection of the micro/meso–scale morphology difference between systems loaded with a sparingly soluble drug *versus* systems loaded with a very slightly soluble drug. The matrix loaded with the sparingly soluble drug (L-dopa) forms the highest number of distinguishable matrix regions (layers) during hydration. For this reason it will be described first.

### Layer Formation in Matrices Containing HPMC and L-Dopa

**Fig. 3:**
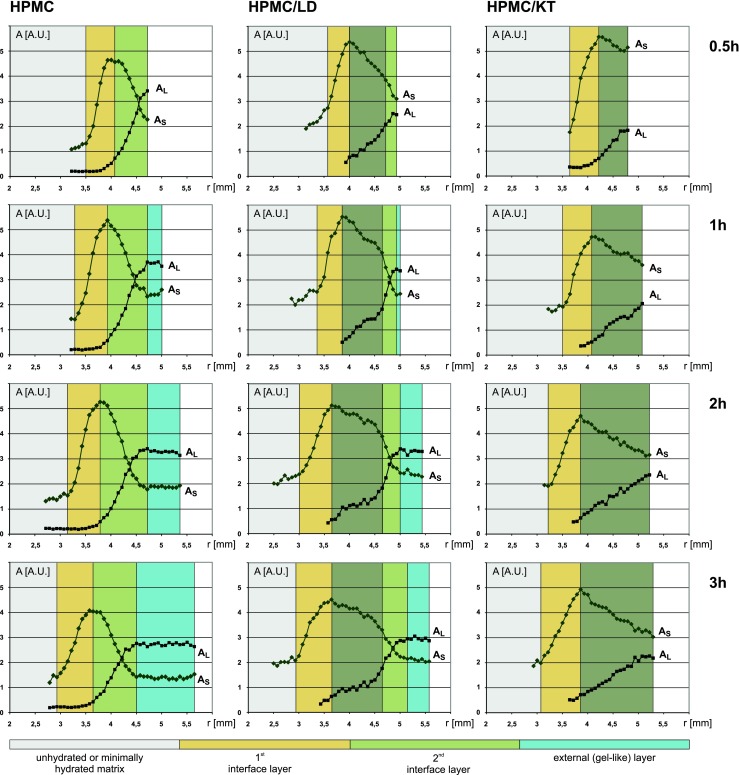
Temporal evolution of the matrices–amplitudes of T_2_ decay envelope components *vs*. tablet radius at 0.5, 1, 2 and 3 h of hydration for HPMC (*left*) HPMC/LD (*centre*) HPMC/KT (*right*) tablets. A_S_–amplitude of the shorter component, A_L_–amplitude of the longer component. The following layers are marked (going outwards from tablet centre): *gray*–minimally hydrated matrix (*solid like*), *yellow*–the first interface layer, *deep/light green*–the second interface layer, *blue*–firm “gel-like” layer.

As it was stated above, the most complex layer formation inside the HPMC matrix loaded with drug can be observed in T_2_/PD profiles obtained for HPMC/LD at hydration times of 0.5–3 h (see Fig. 3). A representative example is presented for samples hydrated for 2 h in Fig. 2.

The layers in the radial profiles were identified due to the criteria described in details in our previous article (13). Identification of the different layers was based on the characteristics of the parameter profile behavior (i.e. increasing, decreasing or constant). An additional feature observed here is that the second interface layer identified in the pure HPMC matrix can be divided into two sublayers utilizing the discontinuity in the T_2L_ profile for the HPMC/LD system. In particular we observe:

1. The second interface layer, the first sublayer (marked dark green)
  Criteria of selection: two components exist; their parameters change slightly across this layer.
  Detailed description: The longer T_2_ component, characterized by A_L_ and T_2L_, appears. Across this layer the amplitude of the longer component (A_L_) increases slightly in linear manner away from the center of the tablet. Moderate slope ranged from 1.64 A.U./mm at 0.5 h to 0.67 A.U./mm at 3 h of hydration. At the same time the amplitude of the shorter component (A_S_) decreases linearly. The slope for A_S_ was similar as for A_L_ but of opposite sign. This phenomenon occurs while T_2_ values of both components (T_2L_ and T_2S_) increase. The end of the sublayer is associated with a distinct discontinuity in the T_2L_ profile and is preceded with a slope change in A_L_ and A_S_ profiles.
2. The second interface layer, the second sublayer (marked light green)
  Criteria of selection: two components exist; their parameters change over a wide range but in a systematic manner across this layer.
  Detailed description: It starts at the position of a discontinuity in the T_2L_ profile. Across this layer the amplitude of the longer component (A_L_) increases linearly away from the center of the tablet. At the same time the amplitude of the shorter component (A_S_) decreases. The slopes of A_L_ and A_S_ change in the second sublayer and are steeper than in the first sublayer. For example, the A_L_ slope ranges from 4.95 A.U./mm at 1 h to 3.57 A.U./mm at 3 h of hydration and is 3–6 times steeper than in the first sublayer. This phenomenon occurs while T_2_ values of both components (T_2L_ and T_2S_) increase. “Amplitude crossing” occurs approximately when the T_2_ value of the shorter component (T_2S_) reaches a plateau. The amplitude of both components reach a plateau when the T_2_ value of longer component (T_2L_) reaches its plateau.

**Fig. 4:**
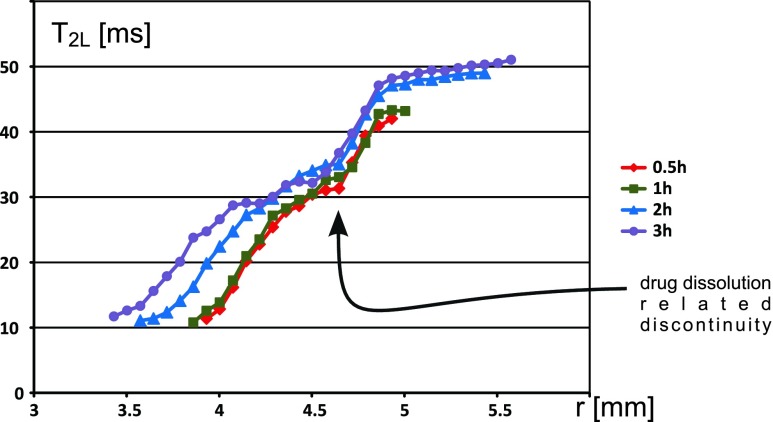
Profiles of longer T_2_ component along the radius of the tablet in HPMC/LD formulation at four hydration times.

The key feature is a distinct discontinuity of the T_2_ profile of the longer T_2_ component (T_2L_) (see Figs. 2 and 4). It is accompanied with slope change in corresponding PD profiles (A_L_, A_S_) (see Figs. 2 and 3). This suggests a rapid spatial change in matrix properties. It appears only for the matrix with sparingly soluble drug. For this reason it can be associated only with drug dissolution process inside the matrix. The discontinuity can be observed for all registered hydration times, and for this particular matrix the position of the discontinuity does not change substantially with time. Therefore, the second sublayer (light green) contains a sufficient amount of dissolved drug to have the same character of spatial changes like the second interface layer in the pure HPMC system and consequently to form an external “gel-like” layer.

The threshold initiating a slope change in the A_L_ profile is under 2 AU for samples measured at 1, 2 and 3 h. But at the hydration time of 0.5 h, a slope change is hard to observe despite the higher A_L_ (up to 2.51 A.U.). T_2_ profile shows a discontinuity even at 0.5 h of hydration, however, the “gel-like” (diffusion) layer formation is of negligible size (time factor for “gel-like” layer formation is needed).

Key differences between matrix hydration of HPMC/LD and pure HPMC can be summarized briefly as:

- in the system loaded with LD, the 2nd interface region is thicker than in a pure HPMC matrix, at the expense of the external “gel-like” (diffusion) layer.
- sublayers in the 2nd interface layer appear and existence of these layers can be associated with the presence of undissolved and dissolved drug substance in the matrix.

### Layer Formation in Matrices Containing HPMC and Ketoprofen

As it can be seen from Fig. 2, the T_2_/PD pattern in the hydrated HPMC/KT system is a subset/part of that registered for HPMC/LD formulations. Starting from the position of full hydration (at maximum of the A_S_) the longer component, characterized by A_L_ and T_2L_, appears (exactly as in the HPMC/LD case). Then, the amplitude of the longer component (A_L_) increases slightly (moderate slope) away from the center of the tablet. At the same time the amplitude of the shorter component (A_S_) decreases in a similar manner. The drug dissolution related effects in the matrix are absent. Amplitude (A_L_) of the longer T_2_ component (water of higher mobility content) for HPMC/KT reaches higher values than threshold for HPMC/LD (2.36 A.U. *vs*. 1.59 A.U. at 2 h of hydration), but neither a discontinuity in T_2_ profile nor a slope change in PD were observed. It can be hypothesized that there are no sufficient conditions to trigger/enable a transition to the “gel like” state. The corollary is that the HPMC matrix with the very slightly soluble drug substance (ketoprofen) is not able to form the most external two regions observed for HPMC with sparingly soluble drug. i.e.

- 2nd sublayer of 2nd interface layer associated with dissolved drug;
- homogeneous external “gel like” layer.

### Properties of External Layer (MRI)

Properties of the external layer in the swelling matrix are a crucial factor for drug release. As a result of different layer formation, the external layers in matrices loaded with sparingly and very slightly soluble drug have different properties (see Fig. 2.) reflected in the dynamic properties of water.

Molecular mobility of water expressed by T_2_ relaxation times of the most external parts of the pure HPMC, HPMC/LD and HPMC/KT matrices are presented in Table I. Additionally, relaxation times at the T_2L_ profile discontinuity for HPMC/LD matrix are also presented in Table I.

**Table I:** T_2_ Relaxation Times of the Hydrated Matrices

	HPMC @ external border		HPMC/LD @ discontinuity		HPMC/LD @ external border		HPMC/KT @ external border	
Time [h]	T_2L_ [ms]	T_2S_ [ms]	T_2L_ [ms]	T_2S_ [ms]	T_2L_ [ms]	T_2S_ [ms]	T_2L_ [ms]	T_2S_ [ms]
0.5	59.4	28.7	31.3	16.3	42.0	22.5	30.8	14.9
1	52.7	28.8	34.6	19.0	43.2	21.8	35.4	17.2
2	46.4	23.4	35.0	20.3	49.0	22.3	34.1	17.7
3	49.7	20.1	32.1	19.8	51.0	22.9	32.2	15.1
Average ± SD	52.1 ± 4.8	25.3 ± 3.7	33.3 ± 1.6	18.9 ± 1.5	46.3 ± 3.8	22.4 ± 0.4	33.1 ± 1.8	16.2 ± 1.2

In case of the HPMC/LD matrix, the external layer is a “gel-like” layer, while for the HPMC/KT system the external layer is the 1st sublayer of the 2nd interface layer with the undissolved drug as can be found in the HPMC/LD matrix. For the HPMC/LD external layer an average T_2L_ was 46.3 ± 3.8 ms while for HPMC/KT matrix was 33.1 ± 1.8 ms. The T_2L_ for HPMC/KT at the external border of the matrix has very similar values as for the HPMC/LD matrix at the T_2L_ discontinuity. It shows that the water molecular mobility at these points is similar. In case of the matrix with very slightly soluble drug (HPMC/KT) the external layer is similar to the 1st sublayer of the 2nd interface layer that was found in the HPMC/LD matrix.

### Properties of External Layers (DSC)

With the DSC method three states of water can successfully be identified: free water, freezable “bound” water and non-freezable “bound” water. The first two states can be distinguished comparing the phase transitions during a heating and cooling runs. The crystallization and melting processes of free water undergo a transition near 0°C while the main exo- and endothermic peaks are shifted toward lower temperatures, representing freezable “bound” water. It is clear that if the interactions between water and the polymer matrix are getting stronger, the crystallization temperatures will be decreasing. The non-freezable bound water absorbed in polymer does not crystallize as a typical exothermic peak, even when the swollen sample is cooled down to −70°C (16).

**Fig. 5:**
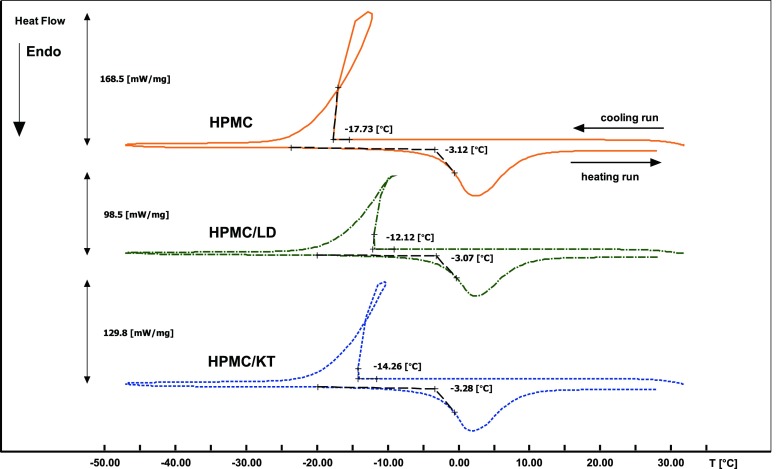
DSC thermograms obtained from the external layers of pure HPMC (*continuous line*), HPMC/LD (*dash-dotted line*) and HPMC/KT (*dashed line*) tablets.

Figure 5 shows the DSC charts of phase transition behavior, contained in the most external layers of pure HPMC, HPMC/LD and HPMC/KT tablets. The observed onset temperatures for crystallization are −17.73, −12.12, −14.26°C and for melting −3.12, −3.07, −3.28°C, respectively. The fact that all those values are far from 0°C indicate that in both types of external layers weak and diversified interactions between water, polymer chains and/or drug take place. Water displays reduced mobility compared to bulk. It suggests that the only existing form of water is freezable “bound” water. The DSC results complement our MRI data. Referring to MRI results, it should be stated that we observed water of higher mobility (characterized by longer T_2_ component) and a less mobile water fractions. Chemical exchange between the polymer water of lower mobility (shorter T_2_ component) and the more mobile fraction is expected in such a system (17). The presence of the components with different T_2_ suggests heterogeneous, spatially separated regions of the matrix occupied by water molecules associated with that components. Moreover, the significant differences in the crystallization onset temperatures lead to the conclusion that the physicochemical state of water in these layers is different.

## DISCUSSION

Physical properties of hydrating polymeric matrices, expressed by formation of specific regions, play a crucial role for the understanding of dosage form behavior during hydration–they ultimately influence drug release from the matrix. But many discrepancies on this topic when using MRI as a method of investigation can be found in the literature. The problem was touched on by Zhang (10) and in the review article by Dorożyński (3). Most of the MRI researchers observe only one hydrated layer (commonly called gel or pseudogel layer) in the matrices loaded with drugs (6,10,11). Some of them observe a second layer denoted as swollen glassy layer (12,18).

Preparation technology, composition and size of the matrix tablets used in our study are similar to these used in the work presented by Tajarobi *et al.* (2009) (6), in which polymer and drug in a 4/6 w/w ratio were compressed into ϕ8 mm tablets. Our results obtained for HPMC matrices with incorporated drugs can clarify their experimental data. In the study by Tajarobi *et al.* in case of an HPMC matrix loaded with DCP (dicalcium phosphate), where DCP is a practically insoluble in phosphate buffer, the drug release follows the erosion of the matrix (the release of the polymer). If, as we suggest, for the matrix with a poorly/insoluble substance “gel-like” (diffusion) layer was not formed, there is no diffusion component in the mechanism of drug release across/from outer layer–in this case the release of active substance should be purely erosion driven. But surprisingly, in contrast to the their release tests or our results, Tajarobi *et al.* (6) did not observe any difference in matrix morphology between the pure HPMC matrix, HPMC with incorporated highly soluble drug (mannitol) and HPMC with insoluble drug by means of MR imaging. For all three systems the detected hydrated region of the matrix is defined/described by using the term “gel”. Similar situation can be observed in article by Viriden *et al.* (7) where matrix properties varied due to HPMC substituent heterogeneity and were manifested by different polymer/drug dissolution profiles. As in previous study authors did not observe differences in matrix morphology using MRI, moreover they showed that dry core disapeared in similar manner. In this context significance of our MRI study stems from the fact that we were able to assess differences in physico-chemical properties of the hydrated matrix.

As a result of the approach presented in previous studies (including MRI), “gel layer thickness” or overall swelling of the matrix was sometimes presented (6,11,19). It was proposed as a performance marker for the pharmaceutical formulations (19). But in the work by Dahlberg, a very cautious approach to the significance of matrix swelling is presented. The authors concluded that the swelling dynamics dominantly controls the drug release kinetics for the “specific system” investigated in their work (20).

Our results confirm that the approach presented by Dahlberg was valid. Physicochemical properties of the swollen region vary with varying solubility of additives (drug). There are no noticeable differences in solvent penetration at consecutive hydration times between all three formulations. But differences in properties of the hydrated matrices in terms of T_2_‘s as well as PD’s and consequently in terms of particular layer formation are unambiguous. It means that overall swelling (or hydration) cannot be used for comparison of different matrix systems. Only similar (“specific” as denoted by Dahlberg) matrices can be compared this way. Moreover, drug release is merely a result of synergy of many factors/mechanisms and cannot be used for deducing mechanisms. While physico-chemical properties between matrices may vary, deep insight into the physical parameters of the hydrating system is necessary for proper matrix dosage form evaluation.

In our review article, (see the box 1 in Dorożyński *et al.* (3)) based on theoretical and experimental literature data, we have presented a multilayer matrix structure including the presence of a drug substance. A similar diagram of the regions and fronts can be also found in the paper by Ferrero *et al.* (21). But so far, there has been no experimental confirmation of a layered structure of HPMC matrices, especially not when using MRI. Only our latest data obtained for pure HPMC matrices (13) have shown that multilayer matrix characterization can be obtained using multiparametric T_2_ relaxometric imaging. In the current study we have shown that HPMC matrices with added drugs also fit the multilayer pattern.

Results of near-infrared (NIR) imaging of the hydration of pure HPMC matrices have been presented by Li *et al.* (2010) (22). The identification of particular components present in each layer was based on a chemometrical analysis. This methodology required dissection of the sample prior to imaging but it provided a large amount of useful information. Their results provide a further confirmation that our approach–identification of five different areas in an hydrating matrix–obtained using multiparametric MR relaxometry, is valid. The layers identified by Li *et al.* (2010) (22) were as follows, starting at the centre of the tablet: a dry matrix core, an area with a high concentration gradient of water (infiltration area), an area of constant water concentration including the phase transition area and the gel layer. Interestingly, the authors also observed that the water concentration increased in time inside the, so-called, dry core of the matrix. Referring to the study by Li *et al.* (22) it can be concluded that the compound transition zones (interface layers) formed between the core of the matrix and the external gel-like outer shell (pure HPMC and HPMC/LD) were detected using MRI methods, for the first time. MRI methods, in this case the multiparametric approach to map spatial distributions of relaxation times, supply much of the information that can be provided by FTIR and NIR methods, but without swelling restrictions or destruction of the sample. MR imaging allows the *in situ* examination of pharmaceutical formulations of any size and shape.

Juxtaposition of results obtained for HPMC with sparingly and very slightly soluble drugs (L-Dopa and ketoprofen, respectively) suggests that the mechanism of region formation is the same in both cases. The key difference is that in the case of HPMC/KT the system is lacking conditions to form a gel-like layer. Gel-like (diffusion layer) layer formation for matrices with incorporated drugs requires dissolution of the drug substance as for the HPMC/LD matrix at 1, 2 and 3 h of hydration (see Fig. 3). Therefore, two factors should be taken into account: “mobile” water concentration and time. For gel formation a sufficient level of water of higher mobility (represented by the amplitude of the longer T_2_ component, A_L_) is required, and this level is dependent on the solubility of the drug substance. For the HPMC/KT system, this level was not reached, even after 3 h of hydration. Considering the time factor, for the HPMC/LD matrix, the gel-like layer does not form immediately after reaching the threshold level of water of higher mobility. It is negligible at 0.5 h of hydration, but appears at 1 h (compare appropriate pictures in Fig. 3).

## CONCLUSIONS

We have introduced a new paradigm in the characterization of hydrating matrices by Magnetic Resonance Imaging methods. High resolution MR relaxometry imaging (T_2_ mapping) as presented in this study, provides new, consistent insight into the hydration of HMPC based polymeric matrices–in this case: pure polymer, and polymer loaded with drugs of different solubility.

For the first time, this drug dissolution related phenomena inside the HPMC matrix, i.e. discontinuity in T_2_ profiles as well as particular front and region formation, were observed by MRI methods. The proposed method allows to characterize micro-mesoscale morphological details and molecular properties of the hydrated part of the matrix in terms of layers. Rich information content (i.e. multilayer structure) was obtained comparing data with previous MRI studies while preserving the possibility to noninvasively image real (various size and shape) pharmaceutical products. We have shown that the morphology of the hydrating HPMC matrix is complex and depends on the solubility of incorporated drug substances. In the most general case (HPMC/LD), the matrix was characterized in terms of a dry core, a minimally hydrated zone, two interface layers (one with two separate sublayers) and an external gel-like (diffusion) layer.

In particular cases layer formation was different:

- In case of HPMC loaded with sparingly soluble L-Dopa all layers/zones were present.
- Pure HPMC matrix does not form the first sublayer in the second interface layer as presented in both drug loaded matrices–this zone is associated with the presence of undissolved drug.
- In case of the HPMC matrix loaded with an very slightly soluble drug substance (Ketoprofen) the system does not form a distinct gel-like layer–only the first two internal interface layers (transition zones) are present.

From a controlled release point of view it could be concluded that drug release from polymeric matrices is merely a consequence of specific matrix formation and the properties of the external part/layer of the matrix. Corollary, nature/morphology of the hydrated part of the matrix should be taken into account when designing (comparing) dosage forms and modeling dosage form behavior.

## ABBREVIATIONS

ATR-FTIR: Attenuated total reflection fourier transform infrared
BLIP_MODES: Broad line imaging package modes
CR: Controlled release
DSC: Differential scanning calorimetry
HPMC: Hydroxypropylmethyl cellulose
KT: Ketoprofen
LD: L-dopa
MR: Magnetic resonance
MRI: Magnetic resonance imaging
MSME: Multi slice–multi echo
NIR: Near-infrared
PD: Proton density
